# Hydration Performance of Magnesium Potassium Phosphate Cement Using Sodium Alginate as a Candidate Retarder

**DOI:** 10.3390/ma15030943

**Published:** 2022-01-26

**Authors:** Yuanquan Yang, Bodong Fang, Guanhua Zhang, Jinbo Guo, Runqing Liu

**Affiliations:** 1 School of Materials Science and Engineering, Shenyang Ligong University, Shenyang 110159, China; yangyuanquan@sylu.edu.cn (Y.Y.); heiyedeguang1621@163.com (B.F.); 2 Liaoning Transportation Planning and Design Institute Co., Ltd., Shenyang 110159, China; lnzgh123@163.com (G.Z.); guojinbo1985@163.com (J.G.)

**Keywords:** magnesium potassium phosphate cement, sodium alginate, compressive strength, setting time, retarder

## Abstract

Retarders are important factors controlling the hydration and properties of magnesium potassium phosphate cements (MKPCs). Boric acid and borax are the most commonly used retarders for MKPC which could control the setting time in a wide range upon changing their content. However, with the increase in borax content, the early strength of MKPC can be reduced, and boron compounds are now included in the EU candidate list of substances of very high concern for authorization, due to their reproductive toxicity. Exploring alternative set retarders to boron compounds is, thus, of significance. This work investigated the effects of a candidate retarder, namely, sodium alginate, on the setting time, mechanical properties, hydration products, and microstructures of MKPC. Sodium alginate presented dramatically retarding effects on MKPCs in the range of 0% to 2% (by mass of water). One percent of sodium alginate by mass of water could extend the setting time of MKPCs from 15 min to 35 min, which presented a better retarding effect than borax (a typical retarder for MKPCs) and produced higher early strength of MKPCs. Adding no more than 1% of sodium alginate did not have a notably adverse effect on the formation of hydration product over the long term, but an unfavorable effect could be found regardless of the sodium alginate content, which could reduce the compressive strength of MKPCs.

## 1. Introduction

Magnesium phosphate cement (MPC) is a kind of inorganic cementitious material [1], formed by hard or dead burnt magnesia, soluble phosphate, mineral admixture, retarders, and water. MPC has been widely used in the fields of rapid repair of concrete pavements, highways, bridge decks, runways, solidification of heavy metals, refractories, and biomedical engineering [2,3], due to its advantages such as fast setting, rapid strength gain, stronger bonding to concrete substrate, and good corrosion resistance. Traditionally, MPC was produced using dead burnt magnesia, ammonium phosphate, and water. However, ammonium gas is released as a reaction byproduct during the MPC fabrication process, which has an unpleasant tangy odor and limits its wider application. To avoid this disadvantage, potassium dihydrogen phosphate is used to replace the ammonium phosphate, and the MPC prepared using potassium dihydrogen phosphate is also called magnesium potassium phosphate cement (MKPC).

The rapid setting of MPC facilitates its rapid strength gain, yet it brings great difficulties and challenges to its practical application in many engineering projects [4,5]. The ability to control cement hydration is, thus, quite crucial. In the literature, several methods have been proposed to slow down cement hydration, including the use of cold water [6], phase change material [7,8], cement dilution with mineral additions, e.g., fly ash [9], metakaolin [10], and wollastonite [11], and the use of different chemicals, such as sodium tripolyphosphate [12], Zn (NO_3_)_2_ [13], aluminum nitrate [4], and boron compounds [14]. However, the retardation mechanisms and their effects on mechanical properties are somewhat different. Lai et al. [13] studied the effect of Zn (NO_3_)_2_ on the early hydration behavior of MPC, and they considered that Zn^2+^ had a significant effect on the hydration process of MPC. It was found that the addition of Zn^2+^ prolonged the final setting time of MPC but reduced the compressive strength of MPC. Li et al. [15] selected low-temperature phase change material CaCl_2_·6H_2_O to adjust the setting time of MPC, and they found that CaCl_2_·6H_2_O can reduce the hydration rate of MPC, prolong the setting time, reduce the content of harmful pores, and improve the strength of MPC, but the content of CaCl_2_·6H_2_O could not exceed 1.5%. Li et al. [16] found that the addition of 20% fly ash slightly extended the setting time of all the MPC samples with or without retarder, and the addition of fly ash could, thus, promote the quantities of magnesium potassium phosphate hexahydrate (main hydration product of MKPC cements). Li et al. [7] applied paraffin/expanded graphite composite phase change material to the MPC system, in which the phase change materials could partially take in the hydration heat from MPC, thus reducing its hydration rate. They also found that adding 8% paraffin/expanded graphite composite phase change material could increase the setting time of MPC by about 1 min, yet lower the strength of MPC. Li et al. [8] also studied the effect of composite phase change materials on setting time and hydration heat of MPC, but found that the composite phase change materials had a minor effect on the hydration temperature of MPC, and their retarding effect was not very obvious. To date, boric acid and borax are the most commonly used retarders to control the setting time in a wide range upon changing their content. Recent studies [14,17] unveiled the underlying retardation mechanisms of boric acid in MPC cements, and it was shown that boric acid slows down the precipitation of the reaction products rather than the dissolution of cement components. In general, the addition of boron compounds at concentrations of a few percent could prolong the setting time of MPC cements from several to tens of minutes [11,12]. However, with the increase in borax content, the early strength of MKPC can be reduced [18], and boron compounds are now included in the EU candidate list of substances of very high concern for authorization, due to their reproductive toxicity [4]. The above mentioned elements emphasize the research significance of exploring alternative set retarders to boron compounds, with an equal or even better retardation effect and improved safety toward humans.

Sodium alginate (C_6_H_7_O_6_Na)n [19] is mainly composed of the sodium salt of alginate; it is usually used as a food thickener, as well as in the dental field [20]. In a previous study, sodium alginate was found to be able to inhibit the growth rate of struvite (main hydration product of MPC) [21]. Sodium alginate was, therefore, tested as a set admixture, replacing borax in our preliminary study, and it presented a notable retardation effect. However, the detailed reaction process on how sodium alginate impacts the setting time, compressive strength, and hydration products of magnesium phosphate cement has not been sufficiently investigated and reported.

Therefore, this work was undertaken with the aim of developing a candidate retarder for MKPC, while giving new insight into the hydration properties of MKPC affected by sodium alginate. Experiments were carried out on MKPC pastes with different w/c ratios and sodium alginate concentrations; specifically, the setting time, strength, microstructure, and hydration products of the MKPC were investigated. The hydration properties of MKPC were investigated using a panel of techniques, including mechanical tests, pH measurement, X-ray diffraction, and SEM analysis.

## 2. Materials and Methods

### 2.1. Materials

Dead burnt magnesia (with a diameter of 74 μm, density of 42 g/cm^3^ and Brunauer–Emmett–Teller (BET) surface area of 230 m^2^/kg), respectively, was prepared by the calcination of magnesite at 1700 °C followed by crushing and grinding through a 200 mesh sieve. Potassium dihydrogen phosphate (KH_2_PO_4_ with purity of 99%) and sodium alginate (SA with purity of 99%) were also used in this experiment. Dead burnt magnesia was obtained from Zhonghao Magnesium Co., Ltd (Haicheng City, Liaoning Province, China). KH_2_PO_4_ was obtained from Sinopharm Chemical Reagent Co., Ltd. (Shanghai, China). Sodium alginate was obtained from Tianjin Beilian Fine Chemical Co., Ltd. (Tianjin, China).

### 2.2. Preparation

The compressive strength and setting time of MKPCs were determined according to GB/T 1346 “Test method for water consumption, setting time, and stability of cement standard consistency” [22] and JC/T 2537-2019 “Magnesium phosphate repair mortar” [23]. Mix proportions are shown in Table 1. Note that an increase in w/c could decrease the compressive strength of MKPCs; however, if the w/c was too small, the hydration of MKPC was not sufficient, resulting in unfavorable strength. The w/c ratios in the range 0.1–0.35 have been frequently used to make cement pastes [5,10,16,24,25,26]. Therefore, the cement pastes (Table 1, samples 1–9) were prepared at three w/c ratios of 0.15, 0.2, and 0.25. To specify the pH evolution of MKPC affected by sodium alginate, cement suspensions (Table 1, Samples A, B, and C) were also prepared according to the mix designs in Table 1, and the pH of the cement suspension was recorded every 3 min with a pH electrode (Seven Multi METTER TOLEDO, S470-K, Zurich, Switzerland).

SA concentration refers to the mass percentage of sodium alginate in the water required by MKPCs. To prepare SA solution, SA and distilled water (if there is no special instruction, the distilled water used in this test was placed at room temperature for reuse) were firstly weighted and then co-mixed for 3 min, before standing for 25 min until completely dissolved. Afterward, dead burnt magnesia and KH_2_PO_4_ were weighed according to Table 1 and stirred at a low speed for 1–2 min. Then, the prepared SA solution was placed into the solid mixture before quickly stirring for another 2–3 min. Finally, the paste was quickly poured in 2 cm × 2 cm × 2 cm mold and demolded after 1.5 h. Then, the specimens were placed in a curing room at a temperature of 23 ± 2 °C and relative humidity of 50 ± 5% to the specified age.

### 2.3. Characterization

The final setting time values of the prepared MKPC pastes were determined by Vicat needle testing according to ASTM C191-13 [27]. To determine the measurement uncertainty, the setting time test for every mixing proportion was conducted twice.

Following the abovementioned casting protocol, three samples (at every specified age) with dimensions of 2 cm × 2 cm × 2 cm were used for compressive strength measurement at a loading rate of 1 MPa/s and a contact surface area of 2 cm × 2 cm.

Scanning electron microscopic imaging (SEM, Hitachi S-3400N, Tokyo, Japan) was carried out on the MKPCs with or without sodium alginate after 3 h, 1 day, and 28 days curing. A small piece was cut from the center part of a sample, immersed in absolute ethanol for 1 week to remove all free water, and stored at 40 °C for another 2 weeks to remove any remaining ethanol. Then, the small pieces were subjected to SEM analysis. Following the same treatment protocol, a small piece of the sample was ground by hand into powders with a grain <100 μm. Afterward, the powders underwent X-ray diffraction analysis (XRD, Rigaku Ultima IV, Tokyo, Japan) using Cu Kα radiation (a scanning time of 15 min and a 2θ range from 5° to 85°).

## 3. Results and Discussion

### 3.1. Setting Time

**Figure 1:**
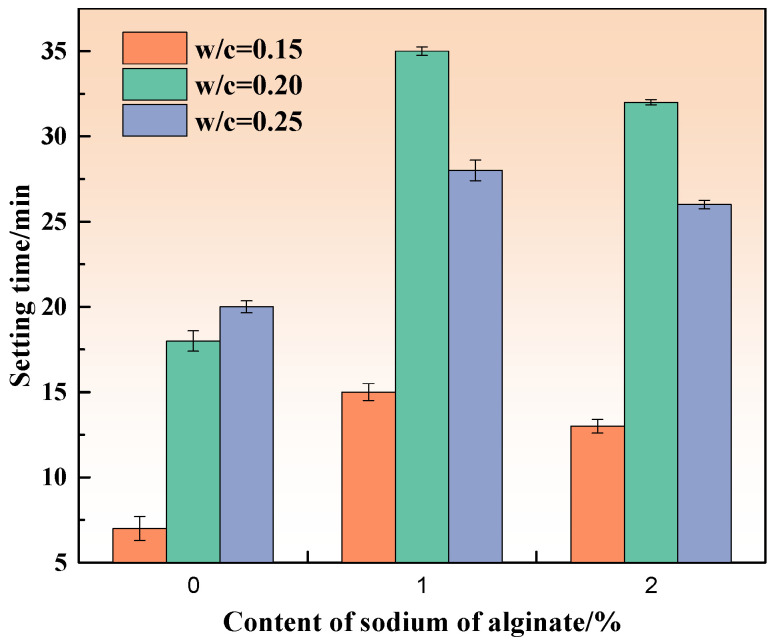
Effect of sodium alginate on setting time of MKPC with different w/c ratios.

Figure 1 shows the setting time of MKPCs with 0%, 1%, and 2% sodium alginate at different w/c ratios.

As displayed in Figure 1, the increase in w/c could positively extend the setting time of MKPCs. The setting time of the MKPCs pastes (reference paste) without sodium alginate increased rapidly from 7 min to about 18 min within a w/c of 0.20, but increased slowly thereafter, which is consistent with the results found by Li et al. [28]. Compared to the reference paste, with the use of sodium alginate could also extend the setting time of MKPCs within a w/c of 0.20, and the setting time was twice that of the MKPCs with a w/c of 0.15. However, the setting time of the MKPCs with sodium alginate decreased rapidly when the w/c was larger than 0.20.

**Figure 2:**
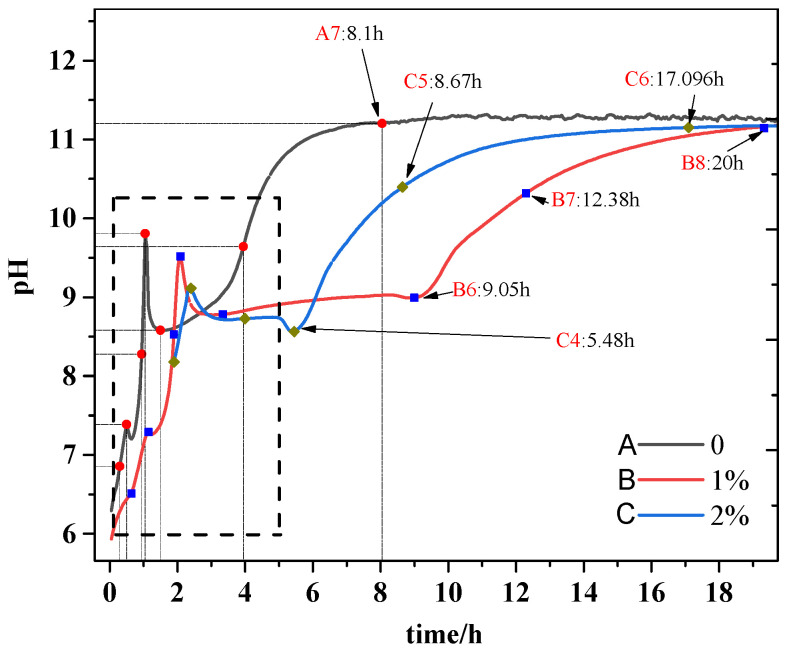
Evolution of pH over time for MPC diluted suspension mixed with different concentrations of sodium alginate.

Interestingly, Figure 1 also presents that sodium alginate could prolong the setting time of MKPCs with sodium content regardless of the w/c ratios. However, the setting time of the MKPC with 2% sodium alginate decreased by 13.3%, 8.5%, and 7.4%, respectively, compared to that with 1% sodium alginate at w/c ratios of 0.15, 0.2, and 0.25. The setting time of the MKPCs with sodium alginate at a w/c of 0.2 presented the best results compared to other w/c ratios. The dissolved ions from sodium alginate could possibly affect the pH of the MKPCs and alter the hydration products, which would change the reaction paths of MKPC and, thus, extend the setting time of the MKPC. To address the effect of sodium alginate on the pH evolution of MKPCs, the evolution of pH over time for an MKPC diluted suspension mixed with different concentrations of sodium alginate was recorded, as shown in Figure 2. It can be seen from Figure 2 that adding sodium alginate could remarkably reduce the pH value of MKPC suspensions at the very early stage (about 2 h). Moreover, the pH evolution of the MKPCs differed greatly, especially when 1% of sodium alginate was incorporated, implying a different reaction pathway. The results demonstrate that the dissolved ions from sodium alginate could possibly affect the pH of the MKPCs, but the detailed reaction mechanism by sodium alginate should be investigated in the future.

It should be noted that further adding sodium alginate (more than 2%) did not substantially extend the setting time of the MKPCs. The results were quite different from boric acid, since the increase in boric acid content could consistently and efficiently increase the setting time of MPC [29]. Higher sodium alginate contributed to a minor improvement of the setting time of MKPC, where the retarding mechanism seemed to differ from conventional retarders such as borax and boric acid for MKPCs. With the increase in sodium alginate content, unfavorable fluidity and unexpected agglomeration phenomena were observed. These were most likely caused by the adsorption and storage capacity of water by the sodium alginate. When dissolved in water, sodium alginate could capture the water molecules during the reaction. When more water is captured, the workability of the MKPC becomes poor, which lowers the quantity of available water for the MKPC hydration reaction.

### 3.2. Compressive Strength

**Figure 3:**
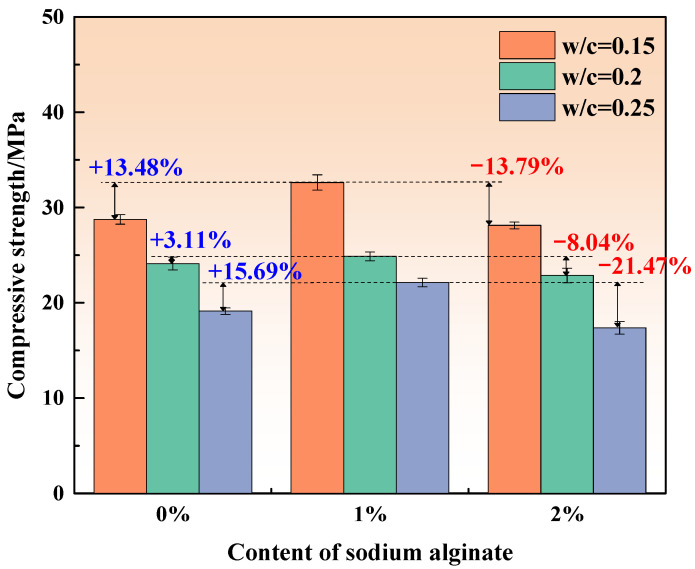
Compressive strength of the MKPCs after 3 h of curing.

**Figure 4:**
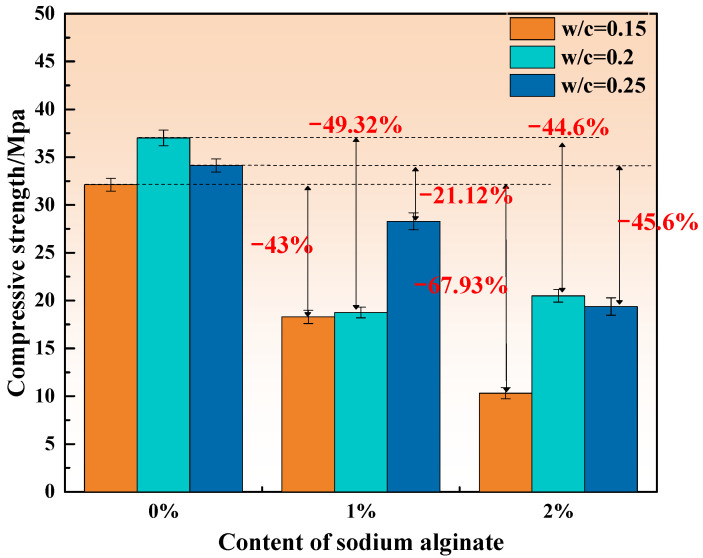
Compressive strength of the MKPCs after 1 day of curing.

**Figure 5:**
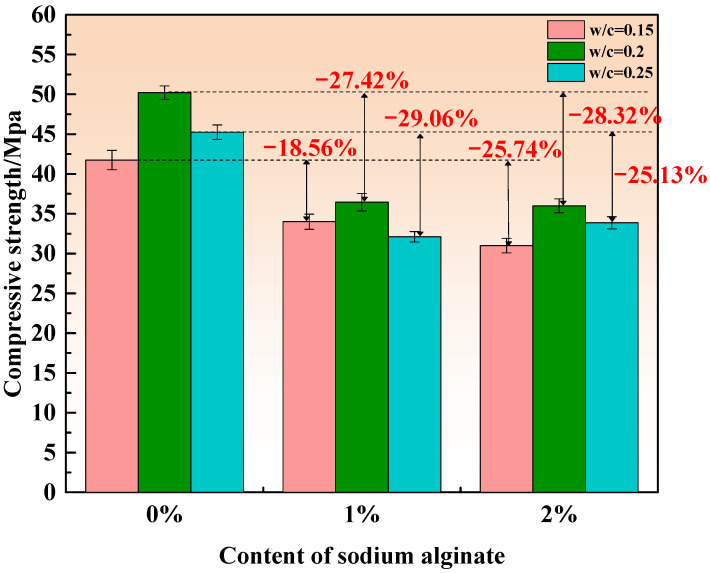
Compressive strength of the MKPCs after 28 days of curing.

Figure 3, Figure 4 and Figure 5 show the compressive strength of MKPCs with different sodium alginate curing for 3 h, 1 day, and 28 days, respectively.

The compressive strength of MKPCs cured for 3 h, as displayed in Figure 3, showed a decreasing trend under the same concentration with the increase in w/c ratios. The amount of water participating the hydration of MKPCs is limited; thus, excessive water could increase the voids of hardened MKPCs and, thus, reduce the compressive strength. This is consistent with the conclusions from Feng et al. [30] and Bi et al. [25]. The strength of the MKPCs increased when the concentration of sodium alginate was within 1%, regardless of the w/c ratio. Under the condition of a 0.15 w/c ratio, the strength reached a maximum, being 13.48% higher than that without sodium alginate. Previous studies [12,31] indicated that the increase in retarder contents such as borax could consistently decrease the compressive strength of MKPC at an early stage; however, the effect of sodium alginate on the compressive strength of MKPC seemingly differed with that of borax. Sodium alginate has a profound water storage capacity, which can encapsulate water molecules. When sodium alginate content is low, it can wrap appropriate water molecules, which can reduce the effective w/c ratio of MKPCs and improve the early strength. Appropriate sodium alginate content leads to less available water, fewer voids, and much denser microstructures for the MKPC at an early stage, thus improving the compressive strength. However, excessive sodium alginate content could result in less available water and poor workability, thereby also reducing the quantities of newly formed hydration products and the early compressive strength.

As displayed in Figure 4, the addition of sodium alginate attenuated the compressive strength of MKPCs cured for 1 day, with the exception of the specimen containing 2% sodium alginate at a w/c ratio of 0.2 (an increase in relation to 1%, but a decrease in relation to the reference value). The water retained by sodium alginate during its early stages is slowly released as the hydration of MKPCs continue. Higher sodium alginate can result in more released water at the later stage of MKPCs. However, released water could alter the formation of hydration products considering the poor water resistance of MKPCs, as well as further dissolve unreacted potassium dihydrogen phosphate, thus forming unexpected voids for hardened MKPCs. Therefore, a higher content of sodium alginate is not recommended, as it could lead to a reduction in the compressive strength of MKPC at the later stage.

Figure 5 shows the compressive strength of the MKPCs cured for 28 days. It can be seen that the compressive strength decreased with the increase in sodium alginate content for the MKPCs regardless of the w/c ratios, with the exception of the specimen containing 2% sodium alginate at a w/c ratio of 0.25. The compressive strength of the MKPCs with 2% sodium alginate and a w/c ratio of 0.25 presented a slightly higher strength gain than the MKPCs with 1% sodium alginate. This demonstrates that, at high w/c ratios, a high volume of sodium alginate could absorb more water, which could release excessive water into the MKPC matrix at the later stage, thus contributing to the rehydration of MKPCs with excessive water.

Sodium alginate can retain excessive water and release it thereafter when the water is in shortage. When the content of sodium alginate is low, it can capture a certain amount of water, resulting in a reduction in the effective w/c ratios of MKPCs and a dense structure, thus improving the early strength. With the progress of hydration, part of the retained water is released slowly. However, part of unhydrated potassium dihydrogen phosphate in the MKPC matrix can be dissolved in the excessive released water, resulting in higher porosity and lower compressive strength of the MKPC matrix. Moreover, the released water may change the pH environment of MKPC, thus altering hydration products and reducing the strength at the later stage.

### 3.3. Hydration Products

**Figure 6:**
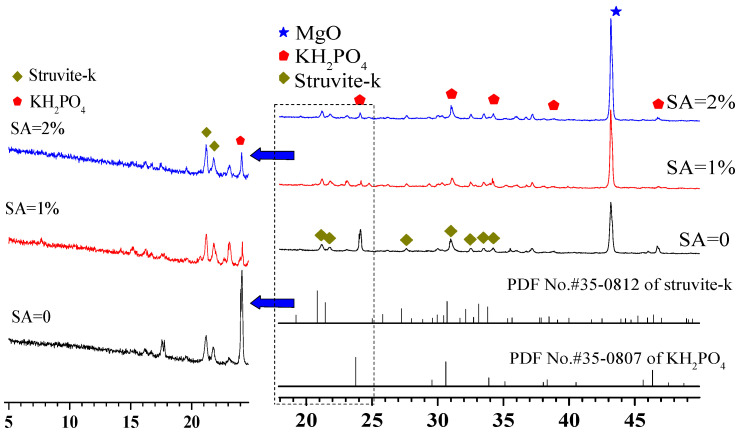
XRD diffraction patterns of MKPC at a w/c ratio of 0.2 after 3 h of curing.

The phase assemblage patterns of the MKPCs at a w/c ratio of 0.2 cured for 3 h were characterized by X-ray diffraction (Figure 6).

Figure 6 exhibits that all MKPCs cured for 3 h had the same phase assemblages regardless of sodium alginate concentration, and all the MKPCs were composed of unreacted MgO, residual KH_2_PO_4_, and newly formed struvite-k. No Na-containing phase was identified when sodium alginate was incorporated. This demonstrated that sodium alginate could not alter the hydration products or, more likely, a not well-crystallized Na-containing hydration product was formed. Sodium alginate seemed to not alter the hydration products of MKPCs, which was quite different to the observations with borax [32], boric acid [14,17] and aluminum nitrate [4].

Interestingly, the dissolution rate of MgO was influenced by the presence of sodium alginate, as more MgO remained upon increasing the sodium alginate content. Specifically, the presence of sodium alginate could lower the dissolution rate of MgO, resulting in more residual MgO. These results coincide with the setting time (Figure 1), where the presence of sodium alginate could extend the setting time of MKPC. However, the presence of sodium alginate led to more residual MgO, but less well-crytallized KH_2_PO_4_, compared to the MKPC that contained no sodium alginate. The presence of sodium alginate seemed to assist the dissolution of KH_2_PO_4_. It is well known that newly formed struvite-k contains both P and K elements; however, the quantity of struvite-k in the MKPCs did not make much difference. This implies that, even though KH_2_PO_4_ was more dissolved, less of it participated in the formation of struvite-k, compared to the MKPC containing no sodium alginate.

**Figure 7:**
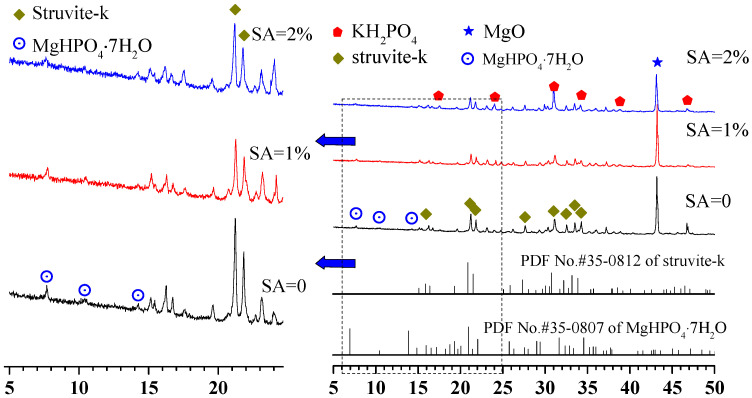
XRD diffraction patterns of MKPC at a w/c ratio of 0.2 after 1 day of curing.

The phase assemblage patterns of the MKPCs at a w/c ratio of 0.2 and cured for 1 day were characterized by X-ray diffraction, as shown in Figure 7. The MKPCs containing sodium alginate exhibited the same phase assemblage as the reference (MKPC without sodium alginate). Struvite-k, residual MgO, unreacted KH_2_PO_4_, and MgHPO_4_·7H_2_O were identified in all of the MKPCs. Note that the quantities of the struvite-k from all the MKPCs cured for 3 h did not differ much. When sodium alginate was added, the formation of struvite-k at 1 day was retarded, as shown in Figure 7, with lower quantities than the reference (MKPC without sodium alginate). Interestingly, even though introducing sodium alginate into the MKPC could retard the formation of struvite-k, subsequently increasing the sodium alginate content for more than 1% could not further lower the quantities of newly formed struvite-k, as shown in Figure 7. The quantities of struvite-k are consistent with the mechanical results of MKPCs at a w/c ratio of 0.2 (Figure 3), where adding sodium alginate could lower the compressive strength of MKPC, and further addition of sodium alginate resulted in a slight increase in compressive strength.

**Figure 8:**
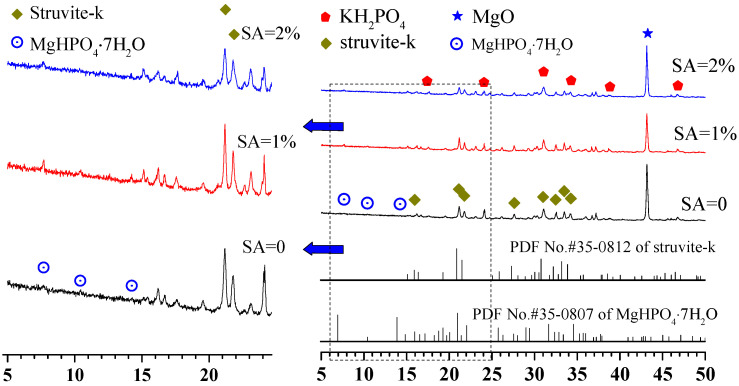
XRD diffraction patterns of MKPC at a w/c ratio of 0.2 after 28 days of curing.

Figure 8 presents the phase assemblage patterns of MKPCs at a w/c ratio of 0.2 and cured for 28 days. The hydration products after 28 days were the same as the MKPCs cured for 1 day. However, the quantities of MgO from the MKPCs with 1% sodium alginate at 28 days were found to be lower than those with 2% sodium alginate and the reference ones. Furthermore, a reverse rule was noticed for the quantities of struvite-k. This suggested that addition of sodium alginate at a content of no more than 1% could contribute to the reaction of the MKPC system, but a reverse effect would be found with more than 1% sodium alginate.

**Figure 9:**
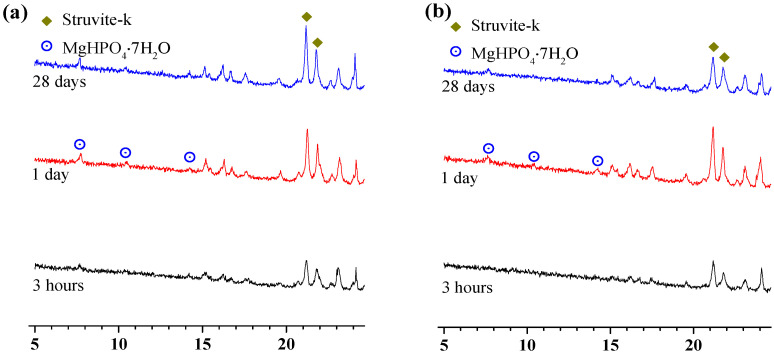
XRD diffraction patterns of MKPC at a w/c ratio of 0.2 at different stages of hydration: (**a**) 1% sodium alginate; (**b**) 2% sodium alginate.

To illustrate the effect of the sodium alginate content on the hydration product of MKPCs, XRD diffraction patterns of MKPC at a w/c ratio of 0.2 are shown in Figure 9 at different stages of hydration. With the progress of hydration, the struvite-k from MKPCs with 1% sodium alginate increased with age. On the other hand, the struvite-k increased first and decreased afterward for the MKPCs with 2% sodium alginate. This suggests that adding no more than 1% of sodium alginate did not have a notably adverse effect on the formation of hydration products over the long term, but an unfavorable effect could be found when sodium alginate exceeded 1%. For the MKPCs with 2% sodium alginate, the struvite-k formed at 1 day tended to be dissolved; therefore, the quantity of struvite-k at 28 days was lower than that at 1 day. This may explain the decrease in compressive strength of the MKPCs at the later stage.

### 3.4. Microstructures

**Figure 10:**
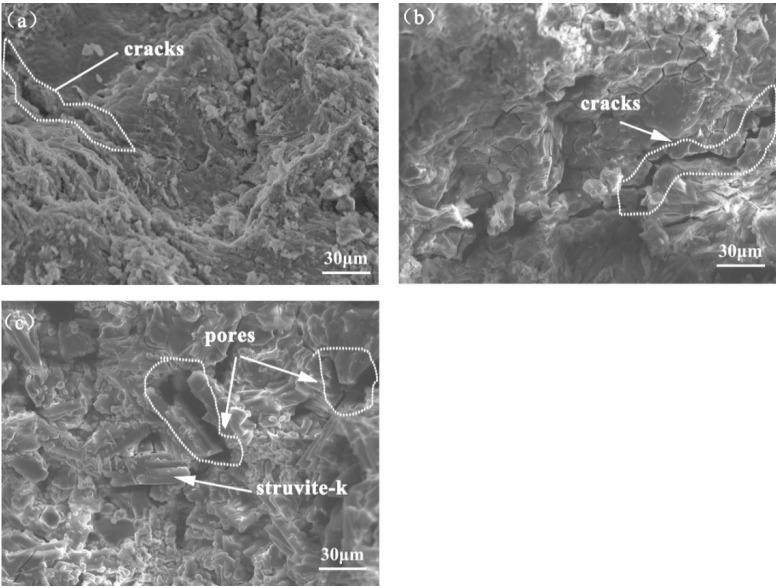
SEM morphologies of the MKPCs at 28 days: (**a**) reference without sodium alginate; (**b**) 1% sodium alginate; (**c**) 2% sodium alginate.

The clearly different micro-morphologies of MKPCs at 28 days are shown in Figure 10. Clear microcracks can be seen for the reference MKPC (Fgiure 10a) and that with 1% sodium alginate (Figure 10b). This could be due to sample dehydration under vacuum [9,33] and/or the compression-induced cracks, because the sample used was collected after the compression test. It can also be seen that the reference MKPC (Figure 10a) and that containing 1% sodium alginate (Figure 10b) presented relatively dense microstructures at 28 days. The magnesia particles were intermixed with struvite-k crystals and the remaining raw materials. Upon increasing the sodium alginate, well-crystallized hydration products were observed (Figure 10c). These results are quite similar to the effect of boric acid content on the properties of magnesium phosphate cement investigated by Ribeiro et al. [29], who found that the increase in retardant concentration favored the formation of more of the struvite phase. Furthermore, adding 2% sodium alginate also led to a large volume of pores in the MKPCs. The pores were supposedly left by the dissolution of struvite-k, as pointed out by the XRD results (Figure 9). This may partially explain why the compressive strength of the MKPCs with 2% sodium alginate decreased after 28 days of curing. Moreover, no new phases such as Na-containing assemblages were clearly observed by SEM analysis; however, the assumption that sodium alginate probably took part in the reaction of MKPCs cannot be excluded. Further evidence should be obtained using, e.g., solid-state NMR (nuclear magnetic resonance) analysis and FTIR spectrometry (Fourier-transform infrared).

## 4. Conclusions

This work was aimed at developing a candidate retarder for MKPC, while giving new insight into the hydration properties of MKPC affected by sodium alginate. The effect of sodium alginate on the setting time, strength, microstructure, and hydration products of the MKPC were investigated, and the main conclusions are as follows:

(1) Sodium alginate presented dramatical retarding effects on MKPCs in the range of 0% to 2% (by mass of water). One percent of sodium alginate by mass of water could extend the setting time of MKPCs from 15 min to 35 min, thus presenting a better retarding effect than borax and producing higher early strength of MKPCs.
(2) The presence of sodium alginate led to more residual MgO at the very early stage, but less well-crystallized KH_2_PO_4_ compared to the MKPCs containing no sodium alginate. The presence of sodium alginate seemed to assist the dissolution of KH_2_PO_4_.
(3) The compressive strength of the MKPCs increased when the concentration of sodium alginate was within 1%, regardless of the w/c ratio. The effect of sodium alginate on the compressive strength of MKPCs seemed to differ with that seen upon introducing borax, which was most likely caused by the adsorption and storage capacity of water by the sodium alginate, in addition to the presence of sodium alginate altering the pH environment of MKPCs.
(4) The addition of sodium alginate at a content of no more than 1% could contribute to the reaction of the MKPC system, but a reverse effect would be found with more than 1% sodium alginate.
(5) No new phases such as Na-containing assemblages were clearly observed by SEM analysis; however, the assumption that sodium alginate probably took part in the reaction of MKPCs cannot excluded, and further evidence should be obtained.

## Figures and Tables

**Table 1:** Mixing proportion.

No.	MgO/g	KDP/g	SA Concentration/%	w/c Ratio
1	108	92	0%	0.15
2	108	92	0%	0.20
3	108	92	0%	0.25
4	108	92	1%	0.15
5	108	92	1%	0.20
6	108	92	1%	0.25
7	108	92	2%	0.15
8	108	92	2%	0.20
9	108	92	2%	0.25
A	108	92	0%	10
B	108	92	1%	10
C	108	92	2%	10

## Data Availability

Not applicable.

## Nomenclature

MPC	magnesium phosphate cement
MKPC	magnesium potassium phosphate cement
SA	sodium alginate
MgO	dead burnt magnesia.
KDP	potassium dihydrogen phosphate.
w/c	water-to-cement (including MgO and KDP) ratio by mass
